# Population Structure and Genetic Diversity of Chinese Honeybee (*Apis Cerana Cerana*) in Central China

**DOI:** 10.3390/genes13061007

**Published:** 2022-06-02

**Authors:** Fang Fang, Xiasang Chen, Jie Lv, Xinyan Shi, Xiaojuan Feng, Zhen Wang, Xiang Li

**Affiliations:** 1 Key Laboratory of Agricultural Animal Genetics, Breeding and Reproduction, Ministry of Education & College of Animal Science and Technology, Huazhong Agricultural University, Wuhan 430070, China; fangfang@mail.hzau.edu.cn (F.F.); lj928027758@163.com (J.L.); 2020302120043@webmail.hzau.edu.cn (X.S.); fengxiaojuan@mail.hzau.edu.cn (X.F.); wangzhen1@mail.hzau.edu.cn (Z.W.); 2 National Center for International Research on Animal Genetics, Breeding and Reproduction (NCIRAGBR), Huazhong Agricultural University, Wuhan 430070, China; 3 Key Laboratory of Pollinating Insect Biology of Agriculture, Institute of Apicultural Research, Chinese Academy of Agricultural Sciences, Beijing 100093, China; 82101211224@caas.cn

**Keywords:** population structure, genetic diversity, environments, *Apis cerana cerana*

## Abstract

Central China has a rich terrain with a temperate monsoon climate and varied natural environments for the Chinese honeybee (*Apis cerana cerana*). However, little comprehensive research on population genetic diversity has been done in this area. A population survey of the structure and genetic diversity of *Apis cerana cerana* in this area is deeply needed for understanding adaptation to variable environments and providing more references for the protection of honeybee biodiversity. In this study, we present a dataset of 72 populations of Chinese honeybees collected from nine sites by whole genome sequencing in Central China. We obtained 2,790,214,878 clean reads with an average covering a depth of 22×. A total of 27,361,052 single nucleotide polymorphisms (SNPs) were obtained by mapping to the reference genome with an average mapping rate of 93.03%. Genetic evolution analysis was presented via the population structure and genetic diversity based on the datasets of SNPs. It showed that *Apis cerana cerana* in plains exhibited higher genetic diversity than in mountain areas. The mantel test between *Apis cerana cerana* groups revealed that some physical obstacles, especially the overurbanization of the plains, contributed to the differentiation. This study is conducive to elucidating the evolution of *Apis cerana* in different environments and provides a theoretical basis for investigating and protecting the Chinese honeybee.

## 1. Introduction

Adaptation to a changing environment is a fundamental problem in evolutionary biology. Understanding the impact of the environment on biological genetic diversity can not only clarify the course of evolution but also provide information on how to protect the ecological environment [1]. Therefore, taking knowledge of genetic resources in a specific natural area will provide references for the strategy design of biodiversity conservation and balancing ecology and economy.

Population structure and genetic diversity are the two main factors leading to ecological and evolutionary dynamics flourishing in the natural environment [2]. Experiment evidence showed that characterization of structural variation has a significant influence on the evolutionary processes of Tibetans’ adaptation to the Qinghai-Tibet Plateau [3]. Except for humans, genetic population structure was also proven to have a regulatory effect on the adaptive evolution of fishes, like sticklebacks [4,5], or other reproducing individuals, including cells, viruses, multicellular organisms and so on [6]. In addition, it has been demonstrated that genetic diversity maintenance is crucial for all species, not just a few domestic species like cultivated plants or domesticated animals [7]. Genetic diversity of the wild animals, insects, and any other special economic animals or crops are also worthy of attention for ensuring all aspects of biodiversity conservation. Some parameters such as expected heterozygous number (He), observed heterozygous number (Ho), nucleic acid diversity (Pi), and polymorphism information content (PIC) can be used to indicate the level of an animal’s genetic diversity efficiency and subdivision of population [8].

Chinese honeybee (*Apis cerana cerana*) is a subspecies and special honeybee resource of *Apis cerana* that evolved over time for long-term adaption to the natural ecological environment in China [9,10]. It mainly nests in distinct habitats of complex topography, divergent climate, and varied flora, resulting in high resistance to changes in the external environment [11,12]. As one of the local advantage species and pollinators with a wide distribution in China [13], *Apis cerana cerana* is a vital component of natural ecosystems that pollinates numerous flowering plants and agricultural crops, providing ecological and financial support to residents [14,15]. Abundant scientific evidence shows that the functional genes of honeybees have excellent adaptability in coping with external environmental changes, ecological diversity and economic profits [16,17,18].

Previous studies on *Apis cerana cerana* have classified it into nine ecological types based on morphometric measurement indicators [19], including Hainan, the Yunnan-Guizhou Plateau, Tibet, Aba, Changbai Mountain, southern Yunnan, North China, South China, and Central China [20]. As more investigators began to study the genetic diversity of *Apis cerana cerana*, our understanding of *Apis cerana cerana* continued to improve until 2015, when the reference genome of *Apis cerana* was published [21]. Whole genome sequencing of a single population can provide a detailed characterization of genetic variation and may clarify the evolutionary development of the population [22]. A comprehensive genomic study of *Apis cerana cerana* in most parts of China indicated the simple hierarchical division. It proposed that physical barriers (such as islands and mountains) were the main obstacles leading to gene exchange, thereby leading to population differentiation [15]. Other studies have reported that *Apis cerana cerana*, even in specific regions, can be highly variable [23,24,25], suggesting that populations from limited geographic areas still have large genetic differences.

Hubei Province is located in Central China, in the middle reaches of the Yangtze River, with a rich terrain and belongs to the temperate monsoon climate. The geographical environment of the area is hilly and mountainous, with elevations ranging from 200 m to 3100 m, and there are more than 150 kinds of nectar source plants, creating excellent regional natural conditions for *Apis cerana cerana* [26,27]. Therefore, surveying the population structure and genetic diversity of *Apis cerana cerana* in this area is deeply needed to understand its adaptation to variable environments and provide more references for protecting honeybee biodiversity in Central China.

## 2. Materials and Methods

### 2.1. Sample Collection

**Figure 1:**
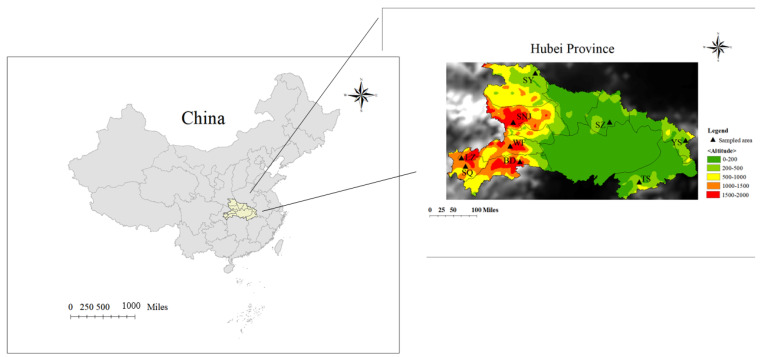
Specific geographic locations of the sampled honeybees in China, where the yellow area represents the sampling site of the representative area of Central China (Hubei Province). Sampling positions in Hubei Province were highlighted in a colorful magnified area map (**right**). Base layer map information comes from Resource and Environment Science and Data Center (page: https://www.resdc.cn/data.aspx?DATAID=200 (accessed on 1 January 2015)).

As experimental materials for this study, 72 worker bees (*Apis cerana cerana*) were obtained from nine different geographic mountain areas in Central China (Table 1 and Figure 1). Sampled honeybees were divided into nine groups of 4 to 10 colonies by geographic names (Table 1). All honeybee samples were stored in a −80 °C refrigerator for further DNA extraction and sequencing.

### 2.2. DNA Extraction

For each bee colony, one worker bee was randomly selected, and total genomic DNA was extracted from its thorax tissue using the Universal Genome DNA Kit (Cat.No.CW0553, ComWin Biotech, Beijing, China) according to the manufacturer’s protocol. After extraction, DNA was treated with RNase A (Cat.No.CW0601S, ComWin Biotech, Beijing, China). The quality and total amount of DNA were measured using Nanodrop 2000 (Thermo Scientific, Wilmington, DE, USA).

### 2.3. Whole Genome Sequencing

The whole genome sequencing of qualified sample genomic DNA was performed on the Illumina Hi-Seq 4000 platform provided by Biomarker Technologies Corporation, Beijing. The DNA was primarily segmented by mechanical interruption (ultrasonic purified). Then the fragment size was selected by agarose gel electrophoresis after the end repairing, dA-tailing and adapter ligation. Eventually, PCR amplification was performed to obtain a 350 bp insert size paired-end library. The established library was first purified and qualified, and finally, the qualified library was sequenced.

### 2.4. Quality Control of Sequencing Reads

The quality of raw reads was validated using FastQC (version 1.11.4) [28]. Adapter sequences, primers, poly-A tails, and low-quality reads were removed with cutadapt [29]. Low-quality data filtering included removing reads with adapter, reads containing more than 10% of N, and reads containing more than 50% bases with a quality value of less than 10 to obtain clean reads for subsequent bioinformatic analysis.

### 2.5. Mapping and Variation Detection

Clean reads were mapped to the reference *Apis cerana* genome (Apis_cerana.ACSNU2.0) with BWA (version 0.7.17) [30]. We statistically processed the information on sequencing depth and genome coverage of each sample for variation detection. Using the Haplotype Caller of GATK (version 4.2.0) [31] to detect the variation of SNP, the annotation of SNP was realized by SnpEff (version 5.1) [32].

### 2.6. Genetic Diversity Analysis

In practical scientific research, when it comes to average correlations between individuals in large genomic datasets, population structure results in systematic patterns visualized using dimension reduction techniques like principal component analysis (PCA) [33], or bayesian clustering methods like STRUCTURE [34,35,36]. PCA, phylogenetic tree, structure and genetic diversity analysis were carried out using vcftools [37] to convert vcf into plink format.

PCA analysis based on the allele frequency distribution among 72 populations was performed to assess the significance of each principal component. PC1 and PC2 were plotted using the ggplot2 package [38] in R 3.6.1 software. To establish the evolutionary relationship among honeybees sampled from nine sites, a neighbor-joining phylogenetic tree was constructed to represent the genetic distances among the population. To accurately identify the ancestral components of the 72 individuals from nine sampling sites, population structure was constructed by a Bayesian clustering program using admixture software [39] to estimate the ancestral composition of each individual with genome-wide unlinked sites. The value of K = 2 to 5 (two–five ancestors) was selected using the admixture model and visualized using R 3.6.1.

### 2.7. Correlations between Environmental Variables and Genetic Diversity

The Mantel test was performed using the Fst matrix and distance matrix with the ade4 software package [40] in R3.6.1. To describe the possible changes in the correlation between the genetic distance and geographical distance, we used PASSaGE (version 2) [41], for Manchester correlation analysis according to the distance. The rank divided the distance matrix into submatrices. The populations within the geographical boundary distance described by each submatrix corresponded to populations with different genetic distances. The average Fst of the set “distance level” was calculated separately to generate a polyline “interference graph” that was combined with the Manchester correlation map for a more intuitive view.

## 3. Results

### 3.1. Genome Sequencing

A total of 72 individual *Apis cerana cerana* from nine geographic units (Hubei Province), including WF, SNJ, YS, LZ, SQ, SY, TS, SZ, and BD (for each, *n* = 4–9), were sequenced. Raw reads of the sequencing data were first filtered to remove the adapter and low-quality sequences, filtered reads with more than a 10% N content, or more than 50% low-quality bases (quality value < 10). After quality control, 2,790,214,878 clean reads in total were generated (Table 2). The average percentage of the sequencing data that yielded a Phred quality score of 30 (Q score > 30) is 91.81% (Table 2). It indicates the probability that a given base is incorrectly called by the sequencer. Subsequently, 150 bp insert size of paired-end reads of clean reads were mapped to the reference genome of *Apis cerana* (Apis_cerana.ACSNU2.0), resulting in 2,7361,052 SNP numbers totally with 22× average coverage depth and an average mapping rate of 93.03% (Table 2).

### 3.2. Population Structure Analysis

**Figure 2:**
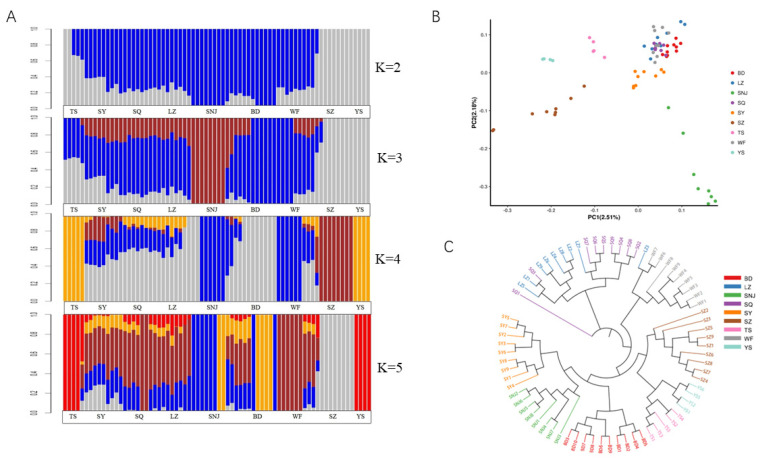
Analysis of the population structure from nine geographic locations. (**A**): Population structure of 72 individuals from different locations. (**B**): Principal component analysis of 72 individuals from different locations. (**C**): Neighbor-joining tree of 72 individuals.

Cluster analysis showed that when the K value was 2, *Apis cerana cerana* in SZ and YS regions were divided into one class. The other regions formed a large group with clear boundaries between regions. When the K value was 3, SZ and YS still formed one group, while the large cluster was split. SNJ formed a cluster. Other regions formed their unique pedigree composition with SNJ, SZ and YS. When the K value was 4, SNJ and WF formed a cluster, TS and YS formed a cluster and SZ formed a cluster, respectively. The other regions (SY, SQ, LZ, BD) formed their unique pedigree composition with these three lineages. The BD and the other three regions showed different degrees of pedigree, and part of the BD area showed a special lineage with clear differences in the area. The result of the PCA scatter plot of principal factor 1 (variation 2.51%) and principal factor 2 (variation 2.18%) further supported the patterns (Figure 2B). When the K value was 5, TS, YS and SZ still formed two clusters, respectively, while SNJ was split into two areas, and the differentiation of SY, SQ, LZ, BD and WF was further emphasized, split into three areas, showing a highly mixed state.

These results were further supported by the phylogenetic tree of the SNP data (Figure 2C). SNJ did not split as a whole and was clustered with SY. TS, YS, and SZ were close. LZ and SQ were clustered into one class. SNJ, SY, BD, TS, YS, SZ, and WF were clustered in a large cluster. These results are consistent with the results of STRUCTURE at K values ranging from 3 to 5. After a comprehensive comparison, we considered that K = 4 was relatively reasonable, and all regions were divided into 5 genetic components; blue region (SNJ, WF), gray region (BD), brown region (SZ), yellow region (YS, TS), and a mixed area of blue, gray, brown and yellow (SY, SQ, LZ).

According to the environmental characteristics of the bee sampling sites (Table 1), we divided the bees collected from the measurement areas into high-altitude type (SNJ, WF), middle-altitude type (BD, SY, SQ, LZ), and low-altitude type (SZ, YS, TS), consistent with the results of STRUCTURE.

### 3.3. Genetic Diversity Analysis

Genetic diversity analysis was performed by calculating MAF, He, Ho and PIC (Table 3). The fixation index (Fst) between populations was calculated to quantify the genetic differentiation (Table 4). The Fst ranged from 0.00706 (LZ, SQ) to 0.05165 (SNJ, YS), with an overall mean of 0.02470, indicating low genetic differentiation. Compared with *Apis cerana cerana* in North China (average Fst = 0.132) [13], *Apis cerana cerana* in Central China was less differentiated at the level of genetic differentiation. The Fst difference between the different regions of the honeybee was small, showing low species diversity at the ecotype level, while the values of Fst were consistent with the overall heterogeneity of the population structure. *Apis cerana cerana* in the central low-altitude regions (SZ, TS, YS) had the highest Fst distribution (average Fst = 0.029965). The average values of Fst in the central high-altitude region and middle-latitude region are 0.02456 and 0.01101, respectively. The Fst 0.051650 between SNJ (high-altitude region) and YS (low-altitude) was moderately differentiated.

The region of Central China (SNJ, WF, BD, SY, SQ, LZ, SZ, YS, TS) showed variable differentiation states, with most being in a low differentiation state, though SNJ and YS were in a moderately differentiated state. Although the genetic differentiation of *Apis cerana cerana* in low-altitude regions (SZ, TS, YS) was higher than in other regions, the Fst average value (0.024696) is lower than 0.05, indicating that the genetic composition of *Apis cerana cerana* in these regions was similar, and the degree of differentiation was low.

The PIC, Ne, Pi, Ho, and He of each SNP locus in the population were separately calculated (Table 3). PIC measured the degree of variation of the population DNA (the overall PIC average was 0.2451). Among these regions, the BD region has the lowest PIC at 0.2334, whereas the average PIC of the YS region was the highest at 0.2812. The populations in lower altitude regions had higher genetic polymorphisms (SZ, TS, YS) with an average PIC of 0.2620, which is consistent with the distribution of Pi. Nucleotide diversity is an indicator of diversity within or between populations. The average Pi values of TS and YS were 0.3614 and 0.3720, respectively, higher than the average (the average Pi = 0.3176). Furthermore, the low-altitude region (TS, SZ, YS) surpassed the mean polymorphic information (Pi = 0.3477 > 0.3176), and there was high differentiation (mean Fst = 0.02456) and high genetic variation (high polymorphism, PIC = 0.2620 > 0.2451).

*Apis cerana cerana* in the lower altitude regions have more biodiversity and heterozygosity than in other areas. Nevertheless, there were differences among heterozygosity (He), variance (PIC), and diversity (Pi) in YS, SZ, and TS, among which YS had higher heterozygosity, variation, and diversity, and while YS and TS belonged to one cluster, SZ belonged to another and had lower heterozygosity, variation, and diversity. A comparison of the STRUCTURE map with a K value of 4 (Figure 2A) revealed that the low-altitude area was divided into another cluster. In addition, we calculated all sampled groups with an average Ho of 0.3368, which was higher than the He of 0.2865. The low-altitude region (YS) has the highest Ho, He, and Pic (0.4126, 0.3477 and 0.2812, respectively), indicating that the population heterozygosity was high, which could be affected by the combined effects of selection, mutation, and genetic drift.

### 3.4. Mantel Test between Apis Cerana Cerana Groups

The correlation between a single or a group of environmental variable factors (such as latitude, longitude, altitude, geographical distance, etc.) was tested. The significant correlation between matrices was analyzed by *p*-value < 0.01 in this study. Mantel test was performed on genetic distance (Fst), environmental factors (Table 5), and geographical distance (Table 6). The results showed that among the seven environmental factors, the genetic distance between populations in nine different geographical regions was positively correlated with the geographical distance, longitude and altitude (*p* = 0.0038 < 0.01, *p* = 0.0015 < 0.01, *p* = 0.0197 < 0.05), but had no significant correlation with other environmental variables (Table 7). The correlation degree between physical distance and genetic distance was r = 0.54243. The correlation degree between altitude and genetic distance was r = 0.32431. These results indicate that geographical distance and location had a significant correlation with the genetic differentiation of *Apis cerana cerana* populations in Central China.

## 4. Discussion

The evolution of species and the generation of diversity are mostly due to environmental changes. Central China has a rich terrain and belongs to the temperate monsoon climate. The geographical environment of this area is plainly and mountainous, with elevations ranging from 200 m to 3100 m, creating a rich source of nectar source plants and excellent regional natural conditions for Chinese honeybee [26,27]. To understand the relationships between genetic differentiation and the environment, this study collected *Apis cerana cerana* from nine regions in Central China and analyzed the population structure and genetic diversity. To obtain as much information as possible about local *Apis cerana cerana*, we included as many collection points as possible in one collection area. The samples were mainly collected from traditional beehives, including crate hives, barrel beehives, and wall hole hives. The domestication process of *Apis cerana cerana* is rather slow, and domestic bees and wild bees often undergo mutual transformation [42]. Therefore, in this study, we believe that different beehive types have a negligible effect on bee society.

The traditional method used 40 morphological indicators to classify *Apis cerana* [13]. These morphological indicators could be classified into several categories, such as color, angle of the right forewing, and body length, to obtain more comprehensive classification results [43]. As the reference genome of *Apis cerana* was published [21], more investigators began to study the genetic diversity of *Apis cerana* [13,44,45]. To analyze the classification, population structure analysis was performed to calculate the degree of mixed blood among individuals if gene exchange has occurred in subpopulations (Figure 2A). When the K value was 4, the STRUCTURE map revealed that SNJ and WF formed a cluster, TS and YS formed a cluster and SZ formed a cluster, respectively. The other region (SY, SQ, LZ, BD) formed its unique pedigree composition with these three lineages. These results were further supported by the PCA analysis and phylogenetic tree (Figure 2B,C), which inferred the differentiation relationships of populations in a low-altitude area separated from populations in high- and low-altitude areas.

Genetic variation is critical for the survival of bees in changing climates. For bees, high genetic diversity at the population level can increase the adaptability of colonies, as bee colonies with higher diversity promote stability and homeostasis [46,47]. To obtain the genetic diversity, we calculated the values of PIC, He, Ho, Pi, and the MAF (Table 3), which can reflect the degree of genetic variation within populations. The Fst value can reveal the genetic differentiation distance between populations (Table 4), which is divided into three levels of genetic differentiation: moderate differentiation (0.05 < Fst < 0.15), larger differentiation (0.15 < Fst < 0.25) and high differentiation (Fst > 0.25). A previous study calculated *Apis cerana cerana* at the subspecies level between different ecological-type populations, for which the average Fst differentiation was 0.162, resulting in high differentiation [15]. In this study, we observed an overall mean Fst value of 0.02470, indicating low genetic differentiation. Compared with *Apis cerana cerana* in a specific region (North China) (average Fst = 0.132) [13], *Apis cerana cerana* in Central China was more lowly differentiated at the level of genetic differentiation. It may be because the geographical distance between populations was limited, and there are no strong physical barriers in this area. There was, however, a special observation that the average Fst between YS and SNJ was 0.05165 (0.05 < Fst < 0.15), reaching the medium differentiation level. We also noticed that the genetic differentiation of *Apis cerana cerana* in low-altitude regions (SZ, TS, YS) was higher than that in other regions. Overall, we speculate that long geographical distances enlarge the genetic differentiation, and the plains are more conducive to gene flow. Previous studies have suggested that honeybees migrate rapidly to areas without strong physical barriers, which can promote gene flow and reduce genetic differentiation [48]. The rapid spread of the African honeybees in European countries can better illustrate the potential for the rapid migration of honeybees [49].

To understand the effect of environmental factors on genetic distance, we calculated the Fst and environmental variable factor of *Apis cerana cerana* in Central China. The correlation between the genetic distance and environmental factors calculated by the Fst is one of the most common methods for assessing the spatial communication of the population structure [50]. Our results showed that geographical distance, altitude, and longitude significantly correlated with the genetic differentiation of *Apis cerana cerana* populations in Central China. We inferred that the mixed terrain of plain and mountain could cause the differentiation. Previous studies have suggested that islands and mountains can form physical barriers that, in turn, cause group isolation [15]. In China, various human activities, such as the loss of agricultural land and urbanization, led to the loss of the habitat of *Apis cerana cerana*, especially in the vast plains of China. However, complex terrains are more resistant to human activities, which could be the reason for the medium differentiation of *Apis cerana cerana* between SNJ and YS. Therefore, we think that the isolation of *Apis cerana cerana* by human urban agglomerations is as important as the physical isolation caused by mountains.

In summary, as a wide-range species, *Apis cerana* shows good potential concerning climate change. Still, stresses such as pesticides, pollutants, pathogens, parasites, and limited flower resources caused by human activities threaten the survival of *Apis cerana* in China [51,52]. This study provides a theoretical basis for investigating and protecting Chinese honeybees by comparing the population structure and genetic diversity of *Apis cerana cerana* from nine geographical regions in Central China.

## 5. Conclusions

This study used genomic data to analyze a wide-range *Apis cerana cerana* species, and provided insights into the differentiation and diversity of the species in Central China. *Apis cerana cerana* in plains exhibited high genetic diversity, and physical barriers (altitude) and distance were the important factors for the differentiation. Human activities, such as urbanization, also greatly impact differentiation. The results of this study contribute to elucidating the evolution of *Apis cerana* in different environments and promote our understanding of how to protect honeybees from current and future challenges.

## Figures and Tables

**Table 1:** Sample information of the *Apis cerana cerana* from nine Central China regions.

Sampling Position	Colony Number	Rearing Mode	Longitude (E)	Latitude (N)	Altitude (m)
WF	9	barrel breeding	110°37′41.61″	30°12′47.39″	1833
BD	10	crate breeding	110°19′20.35″	30°41′48.58″	1208
LZ	9	barrel breeding	108°48′49.95″	30°19′29.23″	1192
SQ	9	barrel breeding	108°56′31.13″	30°4′1.62″	798
SNJ	8	barrel breeding	110°21′55.43″	31°26′1.29″	1464
YS	4	wall hole breeding	115°58′23.89″	30°59′36.18″	399
SY	9	crate breeding	111°6′15.09″	32°57′30.09″	1292
TS	5	barrel breeding	114°19′34.19″	29°34′43.23″	110
SZ	9	barrel breeding	113°24′24.08″	31°26′20.08″	108

**Table 2:** The summary of whole genome resequencing and read mapping results.

Sampling Location	Sample ID	Clean Reads	Q30 (%)	Mapped (%)	Ave-Depth	SNP-Number	Accession Number
SY	SY1	45,364,082	91.48	93.37	25	378,329	SAMN23443044
	SY2	35,194,090	93.20	92.85	20	381,604	SAMN23443045
	SY3	39,560,538	92.29	94.01	23	383,098	SAMN23443046
	SY4	43,425,588	91.00	92.99	25	379,006	SAMN23443047
	SY5	39,156,848	90.57	91.86	22	377,414	SAMN23443048
	SY6	37,591,654	90.80	91.76	21	374,457	SAMN23443049
	SY7	37,202,598	91.63	93.52	22	375,448	SAMN23443050
	SY8	35,763,896	91.73	93.58	21	377,506	SAMN23443051
	SY9	40,626,320	90.93	92.64	23	376,364	SAMN23443052
TS	TS1	49,050,420	92.38	93.37	29	383,607	SAMN23443053
	TS2	42,171,754	93.03	93.08	24	384,279	SAMN23443054
	TS3	43,492,762	92.86	94.28	25	376,482	SAMN23443055
	TS4	44,082,932	90.87	91.91	24	384,675	SAMN23443056
	TS5	41,638,824	92.94	92.85	24	381,053	SAMN23443057
BD	BD1	35,060,548	92.64	91.92	20	378,334	SAMN23443058
	BD2	40,674,004	92.45	92.04	23	383,033	SAMN23443059
	BD3	43,630,338	92.73	92.30	25	383,190	SAMN23443060
	BD4	34,968,120	91.54	92.14	19	375,924	SAMN23443061
	BD5	39,744,324	85.99	90.62	22	376,272	SAMN23443062
	BD6	36,715,404	89.96	91.57	20	376,435	SAMN23443063
	BD7	35,171,312	92.86	92.61	20	382,123	SAMN23443064
	BD8	36,710,574	92.00	93.52	20	377,951	SAMN23443065
	BD9	35,435,816	91.20	92.73	20	376,754	SAMN23443066
	BD10	35,898,376	91.15	93.70	20	382,417	SAMN23443067
SZ	SZ1	34,454,834	87.28	90.80	19	375,325	SAMN23443068
	SZ2	37,622,316	91.01	93.16	22	383,291	SAMN23443069
	SZ3	41,510,880	92.48	92.79	24	381,838	SAMN23443070
	SZ4	34,251,782	92.26	92.58	20	379,841	SAMN23443071
	SZ5	33,690,250	89.19	93.6	20	374,548	SAMN23443072
	SZ6	39,078,406	90.92	93.02	23	375,130	SAMN23443073
	SZ7	43,829,002	91.25	92.31	24	378,748	SAMN23443074
	SZ8	37,989,126	91.58	92.84	21	377,272	SAMN23443075
	SZ9	33,996,270	90.77	92.92	20	376,068	SAMN23443076
SQ	SQ1	37,304,926	92.57	92.21	21	381,208	SAMN23443077
	SQ2	38,685,758	93.03	93.69	22	381,085	SAMN23443078
	SQ3	34,725,076	92.25	94.08	21	380,394	SAMN23443079
	SQ4	34,891,042	92.77	93.41	20	380,657	SAMN23443080
	SQ5	33,717,048	92.93	93.08	19	380,598	SAMN23443081
	SQ6	34,039,492	93.15	94.11	19	381,039	SAMN23443082
	SQ7	34,107,926	90.90	91.77	19	376,245	SAMN23443083
	SQ8	33,680,292	91.45	92.74	18	372,788	SAMN23443084
	SQ9	36,811,506	91.60	93.48	21	379,179	SAMN23443085
LZ	LZ1	45,157,874	90.92	93.42	26	381,808	SAMN23443086
	LZ2	38,943,458	91.03	94.00	23	375,544	SAMN23443087
	LZ3	34,314,032	91.65	90.42	19	377,757	SAMN23443088
	LZ4	40,019,910	92.68	93.60	22	384,578	SAMN23443089
	LZ5	37,750,664	90.86	93.84	21	380,767	SAMN23443090
	LZ6	34,197,778	93.31	93.75	19	379,718	SAMN23443091
	LZ7	35,764,696	90.72	94.67	21	383,465	SAMN23443092
	LZ8	43,571,554	92.81	94.36	26	385383	SAMN23443093
	LZ9	41,338,220	90.69	92.72	24	383,021	SAMN23443094
WF	WF1	34,728,332	90.23	93.40	21	384,993	SAMN23443095
	WF2	35,853,818	91.49	92.29	21	377,900	SAMN23443096
	WF3	45368726	92.98	94.14	27	384,826	SAMN23443097
	WF4	39,606,164	92.60	92.86	23	383,232	SAMN23443098
	WF5	37,016,136	92.73	94.09	22	382,531	SAMN23443099
	WF6	37591406	92.62	93.61	22	384,982	SAMN23443100
	WF7	35,029,444	93.10	93.96	21	384,174	SAMN23443101
	WF8	47,641,082	92.68	93.11	28	385,814	SAMN23443102
	WF9	37,455,550	92.96	93.83	22	382,697	SAMN23443103
YS	YS1	34,459,110	92.31	93.27	20	379,546	SAMN23443104
	YS2	35,456,232	92.76	94.21	21	381,627	SAMN23443105
	YS3	33,847,564	91.76	92.57	22	375,780	SAMN23443106
	YS4	58,232,870	93.03	92.19	32	386,355	SAMN23443107
SNJ	SNJ1	39,745,496	92.83	93.67	24	381,098	SAMN23443108
	SNJ2	57,018,614	92.93	93.63	34	384,589	SAMN23443109
	SNJ3	33,734,502	93.25	93.29	19	379,347	SAMN23443110
	SNJ4	40,561,776	91.05	91.85	23	377,471	SAMN23443111
	SNJ5	42,923,914	93.43	94.12	25	382,094	SAMN23443112
	SNJ6	35,246,256	91.85	94.30	21	375,640	SAMN23443113
	SNJ7	37,636,436	91.44	92.80	22	374,102	SAMN23443114
	SNJ8	37,286,210	91.73	92.64	21	379,204	SAMN23443115
Average	-	38,752,984	91.81	93.03	22		
Total	72	2,790,214,878		-	-	27,361,052	

Note: Description of the column titles contained in the dataset are as follows: Sampling location: Where the samples were collected from Central China. Sample ID: Numbers of the sample population. Clean reads: Number of reads after filtering. Mapped: The percentage of clean reads mapped to the reference genome. Ave-depth: Average coverage depth of sequencing data. Q30: The probability of a given base being called incorrectly is 99.9%. Or we can also think of it as one base out of 1000 is wrong. SNP-number: Number of SNPs.

**Table 3:** Genetic diversity of the *Apis cerana cerana* from nine Central China regions.

	BD	LZ	SNJ	SQ	SY	SZ	TS	WF	YS
MAF	0.1974	0.1996	0.2110	0.1983	0.1987	0.2043	0.2339	0.2007	0.2543
He	0.2828	0.2855	0.2988	0.2840	0.2846	0.2909	0.3257	0.2870	0.3477
Ho	0.3011	0.3049	0.3230	0.3003	0.3025	0.3094	0.3680	0.3085	0.4126
PIC	0.2334	0.2354	0.2452	0.2343	0.2349	0.2393	0.2654	0.2365	0.2812
Pi	0.2962	0.3007	0.3175	0.2991	0.2998	0.3064	0.3614	0.3023	0.3720

Note: He: expected heterozygous number; Ho: observed heterozygous number; Pi: nucleic acid diversity; PIC: polymorphism information content.

**Table 4:** Fst value of the *Apis cerana cerana* from nine Central China regions.

Sampling Position	SY	TS	BD	SZ	SQ	LZ	WF	YS	SNJ
SY	0	0.024002	0.012418	0.018460	0.009056	0.011001	0.012641	0.037860	0.018846
TS	0.024002	0	0.027504	0.027132	0.021796	0.023319	0.025201	0.025201	0.036935
BD	0.012418	0.027504	0	0.027189	0.012009	0.014491	0.015311	0.043329	0.023939
SZ	0.018460	0.027132	0.027189	0	0.023536	0.025641	0.025987	0.037562	0.034366
SQ	0.009056	0.021796	0.012009	0.023536	0	0.007060	0.011214	0.038803	0.021860
LZ	0.011001	0.023319	0.014491	0.025641	0.007060	0	0.013782	0.040189	0.023538
WF	0.012641	0.025201	0.015311	0.025987	0.011214	0.013782	0	0.041681	0.02456
YS	0.037862	0.025201	0.043329	0.0375621	0.038803	0.040189	0.041681	0	0.051650
SNJ	0.018846	0.036935	0.023939	0.034366	0.021860	0.023538	0.024560	0.051650	0

**Table 5:** Information of environmental factors of nine Central China regions.

Sampling Position	Altitude (m)	Annual Minimum Temperature (°C)	Annual Maximum Temperature (°C)	Annual Preipitation (mm)	Longitude (E)	Longitude (N)
SY	292	10	21.01	769.6	111.104	32.958
TS	110	14	23.01	1500	114.326	29.579
BD	1208	14	23	1000	110.322	30.697
SZ	108	12	18	967.5	113.407	31.439
SQ	798	11	18	1450	108.942	30.067
LZ	1192	11	18	1450	108.814	30.325
WF	1833	11	21	1400	110.628	30.213
YS	399	13	23	1403	115.973	30.993
SNJ	1464	9	19	1170.2	110.365	31.441

**Table 6:** Geographic distance between nine Central China regions (unit: km).

Sampling Position	SY	TS	BD	SZ	SQ	LZ	WF	YS	SNJ
SY	0	484.269	261.475	274.807	380.713	363.952	307.734	497.882	181.129
TS	484.269	0	405.175	224.318	523.178	538.449	364.013	198.809	427.997
BD	261.475	405.175	0	305.630	149.914	150.551	61.141	521.081	82.178
SZ	274.807	224.318	305.630	0	453.694	456.169	298.541	233.457	284.364
SQ	380.713	523.178	149.914	453.694	0	31.114	163.273	660.899	207.017
LZ	363.952	538.449	150.551	456.169	31.114	0	175.022	669.051	196.382
WF	307.734	364.013	61.141	298.541	163.273	175.022	0	497.988	136.839
YS	497.882	198.809	521.081	233.457	660.899	669.051	497.988	0	513.726
SNJ	181.129	427.997	82.178	284.364	207.017	196.382	136.839	513.726	0

**Table 7:** *p*-value calculated by Mantel test of Fst matrix of the Chinese honeybee populations and environmental factors.

	Altitude (m)	Annual Minimum Temperature (°C)	Annual Maximum Temperature (°C)	Annual Precipitation (mm)	Longitude (E)	Longitude (N)
*p*-value	0.01978	0.2730	0.1353	0.7359	0.0015	0.07572

## Data Availability

The raw genome sequencing datasets generated during the current study have been submitted to the NCBI Sequence Read Archive (SRA). Accession numbers are listed in Table 2.

## Abbreviations

He: expected heterozygous number; Ho: observed heterozygous number; Pi: nucleic acid diversity; PIC: polymorphism information content; Fst: the fixation index; PCA: a principal component analysis.

## References

[1] Skelly D.K., Joseph L.N., Possingham H.P., Freidenburg L.K., Farrugia T.J. (2007). Evolutionary responses to climate change. Conserv. Biol..

[2] Allen B., Lippner G., Chen Y.T., Fotouhi B., Momeni N. (2016). Evolutionary dynamics on any population structure. Nature.

[3] Quan C., Li Y., Wang Y., Ping J., Zhou G. (2020). Characterization of Structural Variation in Tibetans Reveals New Evidence of High-altitude Adaptation and Introgression. Genome Biol..

[4] Fang B., Kemppainen P., Momigliano P., Merilä J. (2021). Population Structure Limits Parallel Evolution in Sticklebacks. Mol. Biol. Evol..

[5] Kemppainen P., Li Z., Rastas P., Löytynoja A., Fang B., Yang J., Guo B., Shikano T., Merilä J. (2021). Genetic population structure constrains local adaptation in sticklebacks. Mol. Ecol..

[6] Nowak M.A., Tarnita C.E., Antal T. (2010). Evolutionary dynamics in structured populations. Philos. Trans. R. Soc. Lond..

[7] Laikre L., Hoban S., Bruford M.W., Segelbacher G., Vernesi C. (2020). Post-2020 goals overlook genetic diversity. Science.

[8] Peixoto M.G.C.D., Carvalho M.R.S., Egito A.A., Steinberg R.S., Bruneli F.Â.T., Machado M.A., Santos F.C., Rosse I.C., Fonseca P.A.S. (2021). Genetic Diversity and Population Genetic Structure of a Guzerá (*Bos indicus*) Meta-Population. Animals.

[9] Smith D.R., Villafuerte L., Otis G., Palmer M.R. (2000). Biogeography of *Apis cerana F.* and *A. nigrocincta Smith*: Insights from mtDNA studies. Apidologie.

[10] Radloff S.E., Hepburn C., Hepburn H.R., Fuchs S., Hadisoesilo S., Tan K., Engel M.S. (2010). Population structure and classification of *Apis cerana*. Apidologie.

[11] Rol A., Dharam P. (2013). Asiatic Honeybee Apis Cerana.

[12] Zhao H., Li G., Guo D., Wang Y., Xu B. (2020). Transcriptomic and metabolomic landscape of the molecular effects of glyphosate commercial formulation on Apis mellifera ligustica and *Apis cerana cerana*. Sci. Total Environ..

[13] Li Y., Chao T., Fan Y., Lou D., Wang G. (2019). Population genomics and morphological features underlying the adaptive evolution of the eastern honey bee (*Apis cerana*). BMC Genom..

[14] Hou C., Li B., Luo Y., Deng S., Diao Q. (2016). First detection of Apis mellifera filamentous virus in *Apis cerana cerana* in China. J. Invertebr. Pathol..

[15] Chao C., Wang H., Liu Z., Xiao C., Tang J., Meng F., Wei S. (2018). Population Genomics Provide Insights into the Evolution and Adaptation of the Eastern Honey Bee (*Apis cerana*). Mol. Biol. Evol..

[16] Meng J., Wang L., Wang C., Zhao G., Guo X. (2021). AccPDIA6 from *Apis cerana cerana* plays important roles in antioxidation. Pestic. Biochem. Physiol..

[17] Zhao G., Zhao W., Cui X., Xu B., Liu Q., Li H., Guo X. (2021). Identification of an MGST2 gene and analysis of its function in antioxidant processes in *Apis cerana*. Arch. Insect Biochem. Physiol..

[18] Fan W., Li G., Zhang X., Wang Y., Li H. (2021). The role of melatonin and Tryptophan-5-hydroxylase-1 in different abiotic stressors in *Apis cerana cerana*. J. Insect Physiol..

[19] Ruttner F. (1989). Biogeography and taxonomy of honeybees/Friedrich Ruttner. J. N. Y. Entomol. Soc..

[20] Hodges J. (1990). The global organization of animal genetic resources. World Congress on Genetics Applied to Livestock Production.

[21] Park D., Jung J.W., Choi B.S., Jayakodi M., Kwon H.W. (2015). Uncovering the novel characteristics of Asian honey bee, *Apis cerana*, by whole genome sequencing. BMC Genom..

[22] Francioli L.C., Menelaou A., Pulit S.L., van Dijk F., Palamara P.F., Elbers C.C., Neerincx P.B.T., Ye K., Guryev V., Kloosterman W.P. (2014). Whole-genome sequence variation, population structure and demographic history of the Dutch population. Nat. Genet..

[23] Xu X., Zhu X., Zhou S., Wu X., Zhou B. (2013). Genetic differentiation between *Apis cerana cerana* populations from Damen Island and adjacent mainland in China. Acta Ecol. Sin..

[24] Yin L., Ji T. (2013). Genetic diversity of the honeybee *Apis cerana* in Yunnan, China, based on mitochondrial DNA. Genet. Mol. Res..

[25] Fang L., Shi T., Huang S., Yu L., Bi S. (2016). Genetic structure of Mount Huang honey bee (*Apis cerana*) populations: Evidence from microsatellite polymorphism. Hereditas.

[26] Xie C.H. (2012). SRAP Analysis of Genetic Diversity of Riparian Plant Distylium chinense in Hubei Province. Bull. Bot. Res..

[27] Ping L.X., Quan H.Z., Chen F.J., Liang H.W., Lan L.F. (2006). RAPD analysis for the genetic diversity of four populations of Davidia involucrata Baill.in Shennongjia area, Hubei Province. J. Beijing For. Univ..

[28] Andrews S. (2010). FastQC: A quality control tool for high throughput sequence data. Babraham Bioinformatics.

[29] Martin M. (2011). Cutadapt removes adapter sequences from high-throughput sequencing reads. EMBnet J..

[30] Li H., Durbin R. (2009). Fast and accurate short read alignment with Burrows-Wheeler transform. Bioinformatics.

[31] Mckenna A., Hanna M., Banks E., Sivachenko A., Cibulskis K., Kernytsky A., Garimella K., Altshuler D., Gabriel S., Daly M. (2010). The Genome Analysis Toolkit: A MapReduce framework for analyzing next-generation DNA sequencing data. Genome Res..

[32] Cingolani P., Platts A., Wang L.L., Coon M., Nguyen T., Wang L., Land S.J., Lu X., Ruden D.M. (2012). A program for annotating and predicting the effects of single nucleotide polymorphisms, SnpEff. Fly.

[33] Han L., Ralph P. (2019). Local PCA shows how the effect of population structure differs along the genome. Genetics.

[34] Pritchard J.K., Stephens M.J., Donnelly P.J. (2000). Inference of Population Structure Using Multilocus Genotype Data. Genetics.

[35] Falush D., Stephens M., Pritchard J.K. (2003). Inference of Population Structure Using Multilocus Genotype Data: Linked Loci and Correlated Allele Frequencies. Genetics.

[36] Liliana P.H., Yarimar R., Carla S., Christopher P., Ángel C., Lareu M.V. (2013). An overview of Structure: Applications, parameter settings, and supporting software. Front. Genet..

[37] Danecek P., Auton A., Abecasis G., Albers C.A., Anks E.B., Depristo M.A., Handsaker R.E., Lunter G., Marth G.T., Sherry S.T. (2011). The variant call format and VCFtools. Bioinformatics.

[38] Wickham H. (2016). ggplot2: Elegant Graphics for Data Analysis.

[39] Evanno G.S., Regnaut S.J., Goudet J. (2005). Detecting the number of clusters of individuals using the software Structure: A simulation study. Mol. Ecol..

[40] Dray S., Dufour A.-B. (2007). The ade4 Package: Implementing the Duality Diagram for Ecologists. J. Stat. Softw..

[41] Rosenberg M.S., Anderson C.D. (2011). PASSaGE: Pattern Analysis, Spatial Statistics and Geographic Exegesis. Version 2. Methods Ecol. Evol..

[42] Yue-Hui M.A., Gui-Fang X.U., Wang D.Y., Liu H.L., Yang Y. (2003). Study on Dynamic Information of Animal Genetic Resources in China. Agric. Ences China.

[43] Amin E., Brooks S.A., Tomas B., Ellis J.D. (2018). Population genomics and morphometric assignment of western honey bees (*Apis mellifera L.*) in the Republic of South Africa. BMC Genom..

[44] Hao L., Zhou S., Zhu X., Xinjian X.U., Cai Z., Niu Q., Yuan C., Chen D., Zhou B. (2019). Genetic differentiation and genetic diversity analysis of *Apis cerana* in northeast China. J. Northeast Agric. Univ..

[45] Ji Y., Li X., Ji T., Tang J., Zhou X. (2020). Gene reuse facilitates rapid radiation and independent adaptation to diverse habitats in the Asian honeybee. Sci. Adv..

[46] Jones J.C., Myerscough M.R., Graham S., Oldroyd B.P. (2004). Honey Bee Nest Thermoregulation: Diversity Promotes Stability. Science.

[47] Oldroyd B.P., Fewell J.H. (2007). Genetic diversity promotes homeostasis in insect colonies. Trends Ecol. Evol..

[48] Gloag R., Ding G., Christie J.R., Buchmann G., Beekman M., Oldroyd B.P. (2016). An invasive social insect overcomes genetic load at the sex locus. Nat. Ecol. Evol..

[49] Schneider S.S., Degrandihoffman G., Smith D.R. (2004). The African honey bee: Factors contributing to a successful biological invasion. Annu. Rev. Entomol..

[50] Diniz-Filho J.A.F., Soares T.N., Lima J.S., Dobrovolski R., Landeiro V.L., Telles M.P.d.C., Rangel T.F., Bini L.M. (2013). Mantel test in population genetics. Genet. Mol. Biol..

[51] Goulson D., Nicholls E., Botias C., Rotheray E.L. (2015). Bee declines driven by combined stress from parasites, pesticides, and lack of flowers. Science.

[52] Klein S., Cabirol A., Devaud J.M., Ba Rron A.B., Lihoreau M. (2017). Why Bees Are So Vulnerable to Environmental Stressors. Trends Ecol. Evol..

